# The Influence of Drying Temperatures on the Metabolic Profiles and Antioxidant Activity of *Manilkara zapota* Leaves

**DOI:** 10.3390/metabo9100217

**Published:** 2019-10-06

**Authors:** Gloria I. Hernández-Bolio, Rubí E. Dzul-Romero, María G. Maldonado Velázquez, Pedro Zamora Cresencio, Emanuel Hernández-Núñez, Francisco J. Aguirre-Crespo

**Affiliations:** 1 Marine Resources Department, Center for Research and Advanced Studies -National Polytechnic Institute (CINVESTAV-IPN), Unidad Mérida 97310, Mexico; hboliog@gmail.com; 2 Laboratory of Pharmaceutical Biotechnology, Faculty of Biological Chemical Sciences, Autonomous University of Campeche, Campeche 24030, Mexico; rubidr94@gmail.com (R.E.D.-R.); mgmaldon@uacam.mx (M.G.M.V.); 3 Center for Historical and Social Research, Autonomous University of Campeche, Campeche 24030, Mexico; pezamora@uacam.mx; 4 National Council of Science and Technology (CONACYT), Mexico City 03940, Mexico

**Keywords:** ^1^H-NMR, DPPH activity, drying methods, PCA, OPLS-DA

## Abstract

In the present study, the leaves of *Manilkara zapota* (L.) P. Royen (Sapotaceae), an evergreen tree recognized for its medicinal properties in Southern Mexico, were used as a model to study the effect of different drying temperatures on its metabolic profile and therefore, its antioxidant potential. For this purpose, a methanol extraction of leaves dried at room temperature (25 °C) or by heat convection (50, 75 and 100 °C) were compared in terms of drying efficiency, yield of extraction, total phenol content, ^1^H-NMR metabolic profile, and DPPH antioxidant activity. The drying curves enabled the fact to be uncovered that drying efficiency improves with increase of temperature, as does the level of total phenols and antioxidant activity. A metabolomics approach using principal component analysis (PCA) and orthogonal projections to latent structures discriminant analysis (OPLS-DA) of the corresponding ^1^H-NMR profiles allowed the impact of the drying temperature on their metabolic profile to be documented and also, caffeic acid and epicatechin as main secondary metabolites contributing to the antioxidant activity of *M. zapota* to be identified.

## 1. Introduction

One of the simplest and inexpensive methods to preserve the medicinal properties of plants is by drying; also, it is a classical way to start the isolation of natural products [1]. By drying, the plant material becomes easier to handle and less prone to microbial degradation [2], although, it is known that drying temperature may affect the metabolic profile and antioxidant properties of the corresponding extracts [3,4,5]. Heat convection is a process commonly used in the elimination of water vapor during the drying process and the drying curves allow monitoring of the moisture loss as a function of time, thus enabling an estimate of the drying speed of a material [6]. In the context of medicinal plants, the identification of adequate drying conditions ensures the efficiency and drying time as well as the recovery performance of the secondary metabolites of interest.

*Manilkara zapota* (L.) P. Royen (Sapotaceae), also known as chicozapote or sapodilla, is an evergreen tree native to Southern Mexico, Central America, and part of South America [7]. It is mainly cultivated for its fruit, which is highly prized and considered one of the best in Central America [8], although, it is also recognized for its medicinal properties. A decoction of the leaves of *M. zapota* is used as an antipyretic, for treatment of hemorrhage, wounds, and ulcers; other uses include a remedy for diarrhea, intestinal inflammation, lack of appetite, and normalization of blood pressure [9]. Some pharmacological studies have confirmed specific medicinal properties of the leaves such as antidiabetic, antilipidemic, hypoglycemic, and antioxidant activity [10,11,12,13]. While the phytochemistry of the fruit has been explored, reporting the presence of bioactive and novel polyphenols [14], the leaves have only had qualitative studies suggesting the presence of phenols and flavonoids [10] and a single phytochemical study reporting the isolation of lupeol acetate, oleanolic acid, apigenin-7-O-α-l-rhamnoside, myricetin-3-O-α-l-rhamnoside, and caffeic acid [15].

In the present work, we used *M. zapota* leaf extracts as a model to explore the differences in its metabolic profile, and the effect on its antioxidant activity, as influenced by the temperature of the leaf drying. For this purpose, a metabolomics approach using principal component analysis (PCA) and orthogonal projections to latent structures discriminant analysis (OPLS-DA) was performed. The multivariate analysis of the corresponding ^1^H-NMR profiles both allowed the documentation of the impact of drying temperature on the metabolic profile and the detection of the main antioxidant- related metabolites. This method thus contributes to simplifying the pathway to the phytochemical knowledge of a medicinal plant species.

## 2. Results and Discussion

**Figure 1:**
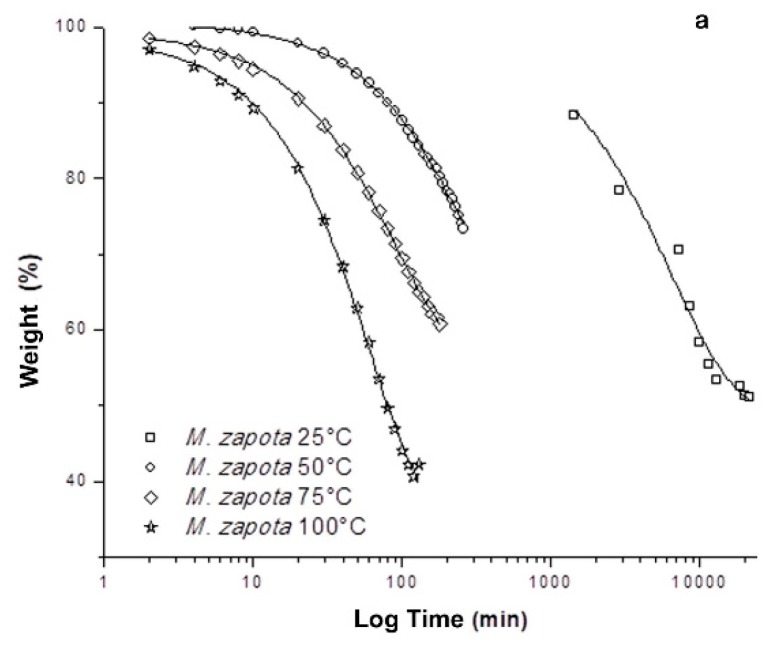
Drying process of *M. zapota* leaves at different temperatures (T = 25, 50, 75 and 100 °C).

For handling and storage purposes, reducing the water content of freshly harvested medicinal plants is imperative [16]. Choosing a drying method includes taking into account features as maximum recovery of metabolites of interest and dry efficiency evaluation in order to obtain a higher yield in a reduced time. For that purpose, a comparison between four drying temperatures of *M. zapota* leaves was carried out. Monitoring of the leaf drying process revealed 100 °C as the most efficient temperature, showing the highest percentage of water loss (57.81 ± 1.48%) in the least time (1 h, 18 min) (Figure 1, Table 1), while the drying at room temperature (25 °C) required at least, 15 days in order to remove 50% of the humidity from the plant material. It is worth mentioning that at 50 °C the efficiency of drying decreases 54.60% when compared to room temperature (25 °C), resulting in a slower drying process and with a lower percentage of water loss.

Poor water removal at 50 °C could be related to stomatal closure in the leaves of *M. zapota*, a situation that prevents transpiration and loss of water in the plant material. With high water stress, the stomatal system closes with increasing temperature, a fact that prevents water loss due to transpiration [17]. It has been reported that in response to drying, the synthesis of abscisic acid (ABA) is increased and its interaction with PYR/PYL/RCAR ABA receptors increases the flow of Ca^2+^ and K^+^ as well as depolarization of the membrane of occlusive cells. These events decrease the turgor pressure, stomatal closure, and reduction of water loss due to transpiration [18]. On the other hand, the use of high drying temperature (75 and 100 °C) favors water vapor pressure as well as protein denaturation involved in the signal transduction of stomatal closure, among other pathways and proteins.

Regarding the methanol extraction of *M. zapota* leaves, it was found that the yield is reduced in a proportional way to the increment of the drying temperature (R^2^ = 0.985, m = –0.31%/°C) (Table 1). The data revealed 100 °C as the temperature with the lowest extraction yield (14.13 ± 0.29%), however, it has the highest simple phenol content (R^2^ = 0.992). On the other hand, it was also the extract with the highest antioxidant activity when tested in the DPPH assay (EC_50_ = 11.04 ± 0.01 µg/mL), thus showing its selectivity in the extraction of antioxidant metabolites (Table 1).

Conversely, few studies analyzing different drying temperatures of plant materials have reported that antioxidant activity decreases with temperature [3,19], and have attributed those changes in the antioxidant activity mainly to the polyphenol content of the plant extracts, which is also reduced with the increment of temperature. It has been shown that polyphenol content is affected by drying [20]. In fact, prolonged exposure at high temperatures reduced 30%, 32%, and 37% of gallic acid, vanillic acid and catequine content, respectively in grape seeds [21]. On the other hand, gallotannins derived from *Caesalpinia spinosa* enhance their antioxidant activity after thermal hydrolysis [22]. In this context, the drying of leaves of *M. zapota* could favor the hydrolysis of gallotannins and increase the content of simple phenols in the extracts as well as the antioxidant activity. Finally, it is reported that thermal stress degrades 11.1%–16.6% of the content of condensed tannins [19] while quercetin exhibits high thermal stability [23]. These facts suggest that simple phenols and other flavonoids could be involved in the antioxidant activity induced by extracts of *M. zapota* leaves.

**Figure 2:**
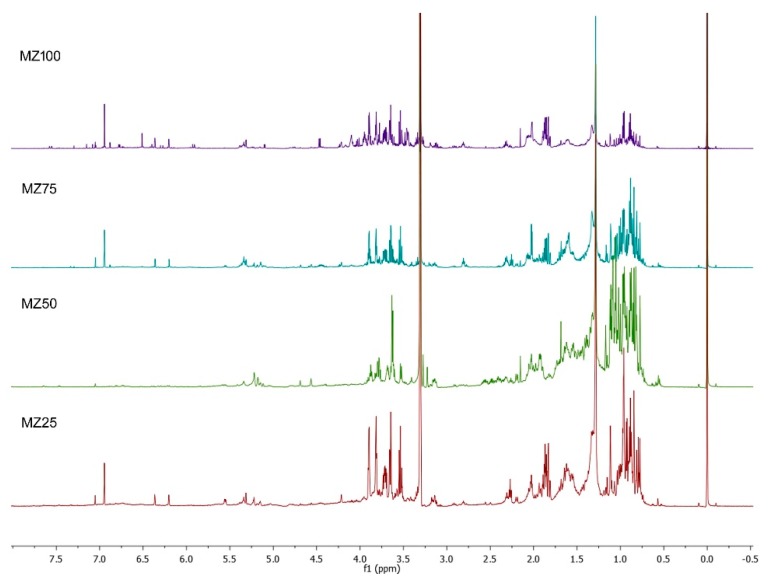
Representative ^1^H-NMR profiles (Region δ 0 to 8) of the MeOH extracts from *M. zapota* leaves dried at different temperatures (T = 25, 50, 75 and 100 °C).

The ^1^H-NMR profiles of methanol extracts from *M. zapota* leaves dried at different temperatures were also compared to detect the changes in the metabolic profile of the corresponding extracts and, furthermore, to explore the possible nature of the antioxidant metabolites. All the extracts showed a high abundance of both aliphatic and carbinolic proton resonances, with the exception of those extracts obtained from leaves dried at 50 °C (Figure 2), which showed a different and simpler profile regarding the carbinolic-proton region and a marked absence of the signals at δ 6.95, 6.36, and 6.21, which could be further assigned to the myricetin (3,5,7-trihydroxy-2-(3,4,5-trihydroxyphenyl)-4H-chromen-4-one) (Table S1). This flavonoid, previously reported from the leaves of *M. zapota* as rhamnoside [15] was thought to be one of the metabolites responsible for the antioxidant activity of the *M. zapota* extract, however, its absence in one of the extracts (MZ50) as shown by the ^1^H-NMR, and the fact that this extract possesses a higher antioxidant activity than MZ25, suggest that myricetin does not have an important contribution for the antioxidant activity of the *M. zapota* leaves extract. (Figure S1).

**Figure 3:**
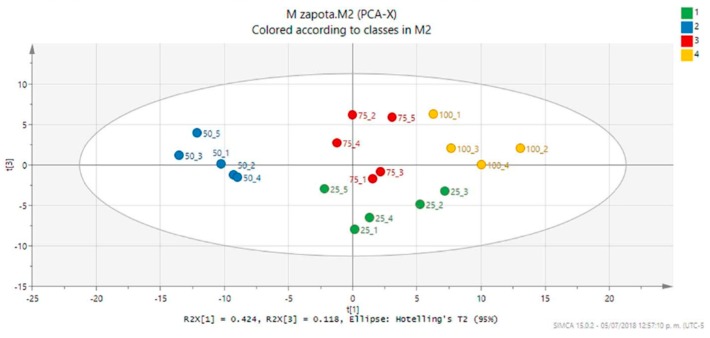
Principal component analysis (PCA) score plot (PC1 vs. PC3, 54.2% of the explained variance) of the *M. zapota* leaf extracts (MZ25, green; MZ50, blue; MZ75, red; MZ100, yellow). The ellipse represents the Hotelling T2 with 95% confidence in the score plot.

The analysis of the PCA score plot (PC1 vs PC3, 54.2% of the explained variance) confirmed the expected grouping according to the temperatures of leaf drying and, as well, showed a clear discrimination of the 50 °C extracts from the others (Figure 3). The PCA ‘contribution plot’ was analyzed in order to detect the signals influencing the discrimination of the 50 °C extracts, showing that these profiles are lacking or with low abundance of signals in the 7.8–5.6 ppm and 4.42–2.68 ppm regions (Figure S1). This change could be related to the poor drying and generation of a high concentration of hydrophilic glycosylated products and therefore, a decrease of organic and methanol-soluble metabolites [24].

**Figure 4:**
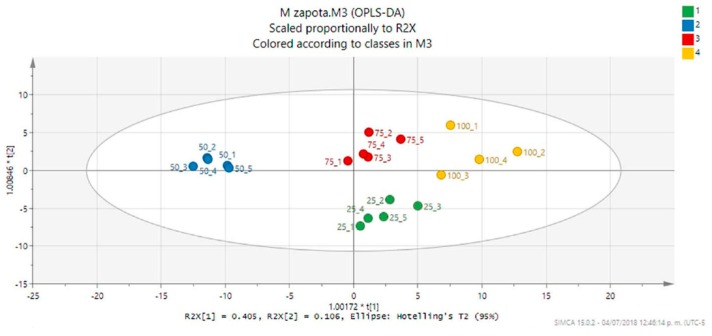
Orthogonal projections to latent structures discriminant analysis (OPLS-DA) score plot (PC1 vs. PC2, 42% of the explained variance) of the *M. zapota* leaf extracts (MZ25, green; MZ50, blue; MZ75, red; MZ100, yellow). The ellipse represents the Hotelling T2 with 95% confidence in the score plot.

**Figure 5:**
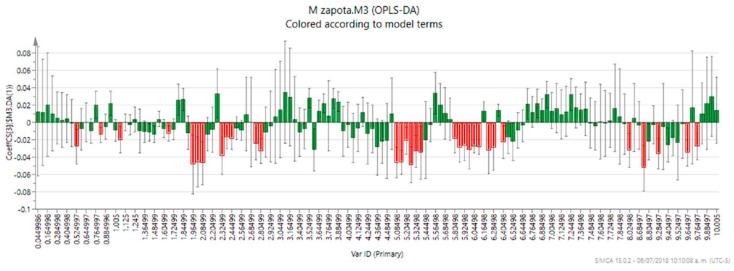
OPLS-DA coefficient plot showing the individual correlation of each bin to the model. Significant bins are shown as red columns.

As stated before, assessment of the DPPH antioxidant activity of *M. zapota* leaves dried at different temperatures revealed 100 °C as the best temperature for the extraction of antioxidant metabolites with a value almost comparable with *C. sinensis* leaf extract (Table 1), a plant well-known for its antioxidant properties. In order to detect the metabolites responsible for the antioxidant activity in the *M. zapota* leaf extracts, an OPLS-DA was carried out using the matrix produced by the PCA and adding as ‘discriminant variables’ the levels of antioxidant activity shown by the extracts. The variance (R^2^) and model predictability (Q^2^) for the OPLS-DA were calculated as 0.827 and 0.659, respectively [25]. While the OPLS-DA score plot showed the characteristic clustering observed earlier in the PCA (Figure 4), the coefficient plot allowed the detection of those bins related to the high antioxidant activity of the *M. zapota* leaf extracts (Figure 5).

In this plot, the coefficients express how strongly the variable Y (e.g., the level of antioxidant activity) is correlated to the systematic part of the X-variables (e.g., spectral bins), meanwhile, the error bars indicate the confidence intervals of the coefficients. If the coefficient is significant, the confidence interval does not include zero. Therefore, 32 bins in total were identified as related to the activity by the analysis of this plot. The analysis of the activity-related bins led to the successful assignation of two antioxidant metabolites as follows: the signals at δ 5.92, 5.86, 2.80, and 2.74 could be assigned to epicatechin, while the signal at δ 6.28 was assigned to caffeic acid.

The complete assignation of the ^1^H-NMR signals corresponding to antioxidant metabolites was carried out by analysis of their ^1^H, ^13^C and bidimensional (^1^H-^1^H COSY, ^1^H-^13^C HSQC, ^1^H-^13^C HMBC and TOCSY) NMR data, as well as comparison with data reported in the literature (Tables S2 and S3, respectively). Epicatechin and caffeic acid, phenolic metabolites that are widely distributed among the plant species, are well-known for their antioxidant effects, having a reported DPPH trolox equivalent activity of 2.7 and 2.2 mM, respectively [25]. Their high antioxidant activity (Table 1) thus validates the model created by OPLS-DA and confirm its proficiency to detect the antioxidant components from *M. zapota*. Regarding the contribution of these phenolic metabolites to the antioxidant activity of *M. zapota*, their absence in the extracts of leaves dried at 25, 50 and 75 °C, explains to a large extent the high level of antioxidant activity of the 100 °C dried leaf extract, however, the presence of unassigned, less abundant signals, suggests that other metabolites can be also displaying some level of antioxidant activity [26]. On the other hand, the fact that some signals corresponding to the antioxidant metabolites were not detected as activity-related can be explained by the overlapping of ^1^H-NMR signals, although, further approaches including *J*-resolved and 2D-NMR metabolomics methods can be further applied [27,28] in order to clearly detect the signals from the individual metabolites. Additionally, the information obtained in studies as the current investigation may contribute to dereplication of plant extracts by the identification and removal of known products in order to detect the minor abundant bioactive metabolites, activities which are currently underway.

The analysis of the ^1^H-NMR profiles of *M. zapota* by such a metabolomics approach allowed the documentation of the impact of drying temperature on their metabolic profile and the identification of caffeic acid and epicatechin as main secondary metabolites contributing to antioxidant activity exerted by methanolic extracts derived from leaves of *M. zapota*. Thus, this represents an efficient, rapid and reliable technique to begin the phytochemical study of medicinal plants and their preservation.

## 3. Materials and Methods

### 3.1. General Experimental Procedures

Deuterated methanol (MeOH-d_4_), trimethylsilane (TMS), gallic acid, and 1,1-diphenyl-2-picrylhydrazil (DPPH) were purchased from Sigma-Aldrich Co. (St. Louis, MO, USA), *Camellia sinensis* was purchased from Badia Spices, Inc. (Doral, FL, USA) and MeOH was analytical grade obtained from a local source. ^1^H-NMR experiments were conducted at 25 °C using a Varian 600 MHz AR Premium Compact (Varian-Agilent, Santa Clara, California, U.S.).

### 3.2. Plant Material and Drying

Fresh leaves of *M. zapota* were collected in March 2017 near Campeche, Campeche, México. A voucher specimen was identified taxonomically by MSc. Pedro Zamora Cresencio and deposited at the UACAM-herbarium under registration number 004727.

Four groups of leaves were processed individually to assess the influence of drying on the metabolic profiles and antioxidant activity of *M. zapota*. A first batch of leaves was room temperature dried (T = 25 °C; m = 15.24 ± 0.23 g) while the remaining groups were oven dried at different temperatures (T = 50 °C, m = 2.21 ± 0.29 g; T= 75 °C, m = 2.17 ± 0.23 g; T = 100 °C, m = 2.56 ± 0.07 g, respectively) using a thermobalance (Mettler-Toledo^®^). The loss of humidity and weight as a function of time were registered until the attainment of a constant weight. An additional analysis of the drying process was carried out by a sigmoidal and non-linear mathematical model. The weight of leaf samples and the loss of humidity are expressed as the mean ± standard error of three experiments with three replicates each (*n* = 9).

### 3.3. Preparation of Plant Extracts

A portion (1 g) of ground, previously dried *M. zapota* leaves was extracted three times by maceration with 28 mL of MeOH (24 h at 25 °C). The combined extractions of each group were concentrated in vacuo at 40 °C (Büchi) to produce the corresponding extracts MZ25, MZ50, MZ75, and MZ100. The yield extraction is expressed as the mean ± standard error of three experiments with three replicates each (*n* = 9).

### 3.4. Total Phenol Content

A calibration curve for gallic acid (GA, 1.04–8.2 µg/mL) was prepared. Briefly, 0.4 mL of GA solution or extract (10 µg/mL) was added to 0.4 mL of Folin-Ciocalteu reagent in 1 mL of distilled water and incubated at room temperature in the dark for 20 min. The reaction was stopped with 1.6 mL of 10% Na_2_CO_3_ solution and the absorbance was recorded at 765 nm. Total phenol content in the methanol extracts was expressed as equivalents (mg/g) of gallic acid present in the plant material [5].

### 3.5. DPPH Assay

The DPPH reducing activity of the *M. zapota* leaf extracts was determined following the method reported by ref. [29] with slight modifications. Briefly, 200 µL of each extract (1–50 µg/mL) was added to 1.8 mL of a 0.1 M DPPH solution in MeOH and vortexed. After 30 min of incubation in the dark, the absorbance at 517 nm was measured. The percentage of remaining DPPH was calculated using the formula: % (DPPH) = [(Abs DPPH sample)_t=30_/(Abs DPPH 0.1 M)_t=0_)] × 100. Spectrometric measurements were made using methanol as a blank. A MeOH extract of *C. sinensis* (1–50 µg/mL), as well as gallic acid (0.03–32 µg/mL), were used as positive control. The assay was run three times for each extract on different days. Finally, from the experimental data and the use of a non-linear model, the potency (EC_50_, [µg/mL]) and efficacy (Emax, (%)) of the antioxidant activity exerted by the extracts was calculated. The antioxidant activity is expressed as the mean ± standard error of three experiments with three replicates each (*n* = 9).

### 3.6. ^1^H-NMR Metabolic Profiles and Multivariate Analysis

A portion (approximately 5 mg) of each extract was dissolved in 600 µL of deuterated methanol (MeOH-*d*_4_) containing 0.05% *v/v* TMS and transferred to 5 mm NMR tubes. The measurements were conducted at a magnetic field of 14.1 T and a ^1^H resonance frequency of 599.77 Hz, using the PRESAT sequence with a total of 128 transients with data collected in 64k data points. The relaxation delay was set 1.0 s and the acquisition time was 3.0 s. The resulting spectra were manually phased, and baseline corrected using Mestrenova 12.0 (Demo version, Mestrelab Research) and referenced to TMS at 0.0 ppm. Additional NMR analyses including ^13^C-NMR, gCOSY, gHSQC, and gHMBC were conducted on a 100 °C dried sample in order to confirm the identification of the detected metabolites.

### 3.7. Statistical Analysis

The weight of leaf samples and the loss of humidity, together with the yield of extraction and the results from total phenol content and antioxidant activity are expressed as the mean ± standard error of three experiments with three replicates each (*n* = 9). The graphics from the drying process and antioxidant activity were obtained in the Origin^®^ 8.0 software, applying ANOVA and significance values of *p* ˂ 0.05. Principal component analysis (PCA) and orthogonal projections to latent structures discriminant analysis (OPLS-DA) were performed using SIMCA 15 software (Demo version, Sartorius-Stedim). NMR spectra were normalized to the TMS signal (δ 0.0), divided into equally sized bins of 0.06 ppm within the region of 10.0 ppm to 0.44 ppm using the ‘average sum’ method in Mestrenova and Pareto scaled. A data matrix was constructed with each row representing a spectrum and each column a variable (e.g., bin). The spectral regions between 3.26–3.36 ppm and 4.5–4.9 ppm (residual solvent signals) as well as uninformative regions as 8.14–8.62 ppm and 8.86–9.16 ppm, were excluded in PCA to yield a total of 141 bins. The binning generated from PCA was used to perform the OPLS-DA. The quality of the model was described by R^2^ (percentage of variation of the training set explained by the Y-predicted components) and Q^2^ (percentage of variation of the training set predicted by the model according to cross validation).

## Figures and Tables

**Table 1:** Experimental results of *M. zapota* leaves dried at 25, 50, 75 and 100 °C.

Drying Temperature (°C)	Water Loss (%) ^a^	Extraction Yield (%) ^b^	Total Phenol Content (mg/g) ^b^	DPPH Antioxidant Activity EC_50_ (µg/mL) ^b^
25	48.94 ± 9.37	3.67 ± 0.03	0.57 ± 0.003	26.41 ± 0.180
50	26.71 ± 3.61	2.88 ± 0.02	0.24 ± 0.004	22.73 ± 0.050
75	39.25 ± 2.00	1.90 ± 0.02	1.21 ± 0.004	17.31 ± 0.010
100	57.81 ± 1.48	1.41 ± 0.03	2.59 ± 0.004	11.04 ± 0.010
*C. sinensis*	-	-	2.96 ± 0.020	9.22 ± 0.060
Gallic acid	-	-	-	2.18 ± 0.01

^a^ Until reaching of constant weight; ^b^ Results expressed as the mean ± standard error of six experiments.

## Supplementary Materials

The following are available online at https://www.mdpi.com/2218-1989/9/10/217/s1, Table S1: Experimental ^1^H, ^13^C-NMR, COSY, HSQC and HMBC data of the putative myricetin and those reported for the myricetin aglycone, Table S2: Experimental ^1^H-NMR data of the putative epicatechin and those reported for the epicatechin, Table S3: Experimental ^1^H, ^13^C-NMR, COSY, HSQC and HMBC data of the putative caffeic acid and those reported for the caffeic acid, Figure S1: Representative ^1^H-NMR profiles (Region δ 5.0 to 8.1) of the MeOH extracts from *M. zapota* leaves dried at different temperatures (T = 25, 50, 75 and 100 °C) showing the characteristic resonances from the detected metabolites.
