# Supplementary material for: The Influence of Drying Temperatures on the Metabolic Profiles and Antioxidant Activity of Manilkara zapota Leaves
Source: Metabolites. 2019 Oct 6;9(10):217. doi: 10.3390/metabo9100217 (PMC6835937; doi:10.3390/metabo9100217)
Supplement: Supplementary file 1 [file metabolites-09-00217-s001.pdf]

metabolites

MDPI

SUPPLEMENTARY MATERIAL

Table S1. Experimental ^{1}H, ^{13}C-NMR, COSY, HSQC and HMBC data of the putative myricetin and those reported for the myricetin aglycone [1].

Position	Myricetin aglycone (CD_3OD, 400 MHz)	Myricetin (600 MHz, CD_3OD)	COSY	Myricetin aglycone (CD_3OD, 100 MHz)	Myricetin (150 MHz, CD_3OD)	HMBC
	^{1}H-NMR			^{13}C-NMR		
5				163.2	163.2	
6	6.21 d (J = 2 Hz)	6.21(J = 2 Hz)	H-8	100.0	99.8	C10, C5, C7
7				166.0	165.8	
8	6.37 d (J = 2 Hz)	6.36 (J = 2 Hz)	H-6	94.8	94.6	C10, C9, C7
9				158.9	158.5	
10				106.0	105.7	
2′, 6′	6.93, s	6.95		110.0	109.6	[a]

[a]Not detected.

Table 2. Experimental ^{1}H-NMR data of the putative epicatechin and those reported for the epicatechin [2].

Position	Epicatechin (400 MHz, CD_3OD)		Epicatechin ^{1}H-NMR (600 MHz, CD_3OD)	
2	4.83	brs	4.85	brs
3	4.19	m	4.22	m
4a	2.86	dd, J = 4.8, 16.8 Hz	2.85	dd, J = 4.7, 16.6 Hz
4b	2.73	dd, J = 2.7, 16.8 Hz	2.73	dd, J = 3.3, 16.6 Hz
5				
6	5.93	d, J = 2.3 Hz	5.91	d, J = 2.3 Hz
7				
8	5.96	d, J = 2.3 Hz	5.94	d, J = 2.3 Hz
9				
10				
1’				
2’	6.99	d, J = 1.7 Hz	6.97	d, J = 2 Hz
3’				
4’				
5’	6.77	d, J = 8.2 Hz	6.73	dd, J = 8.2 Hz
6’	6.81	dd, J = 1.7, 8.2 Hz	6.76	d, J = 2.1, 8.2 Hz

Table 3. Experimental ^{1}H, ^{13}C-NMR, COSY, HSQC and HMBC data of the putative caffeic acid and those reported for the caffeic acid [3].

Position	Caffeic acid (500 MHz, CD_3OD)	Caffeic acid (600 MHz, CD_3OD)	COSY	Caffeic acid (600 MHz, CD_3OD)	HMBC
	^{1}H-NMR			^{13}C-NMR	
α	6.29 *J* = 15.9 Hz	6.29 d, *J* = 16.0 Hz	H- β	115.2	C6
β	7.55 *J* = 15.9 Hz	7.57 *J* = 16.0 Hz	H- α	146.9	Cα, C6
1				127.7	
2	7.07 *J* = 2.0 Hz	7.05 *J* = 2.0 Hz		115.2	C6, C3, C=O
3				146.7	
4				ND	
5	6.81 *J* = 8.2 Hz	6.78 *J* = 8.3 Hz	H-6	116.2	C1, C3
6	6.95 *J* = 8.2, 2.0 Hz	6.95 *J* = 8.3, 2.0 Hz	H-5, H-2	122.9	C3
C=O				170.5	

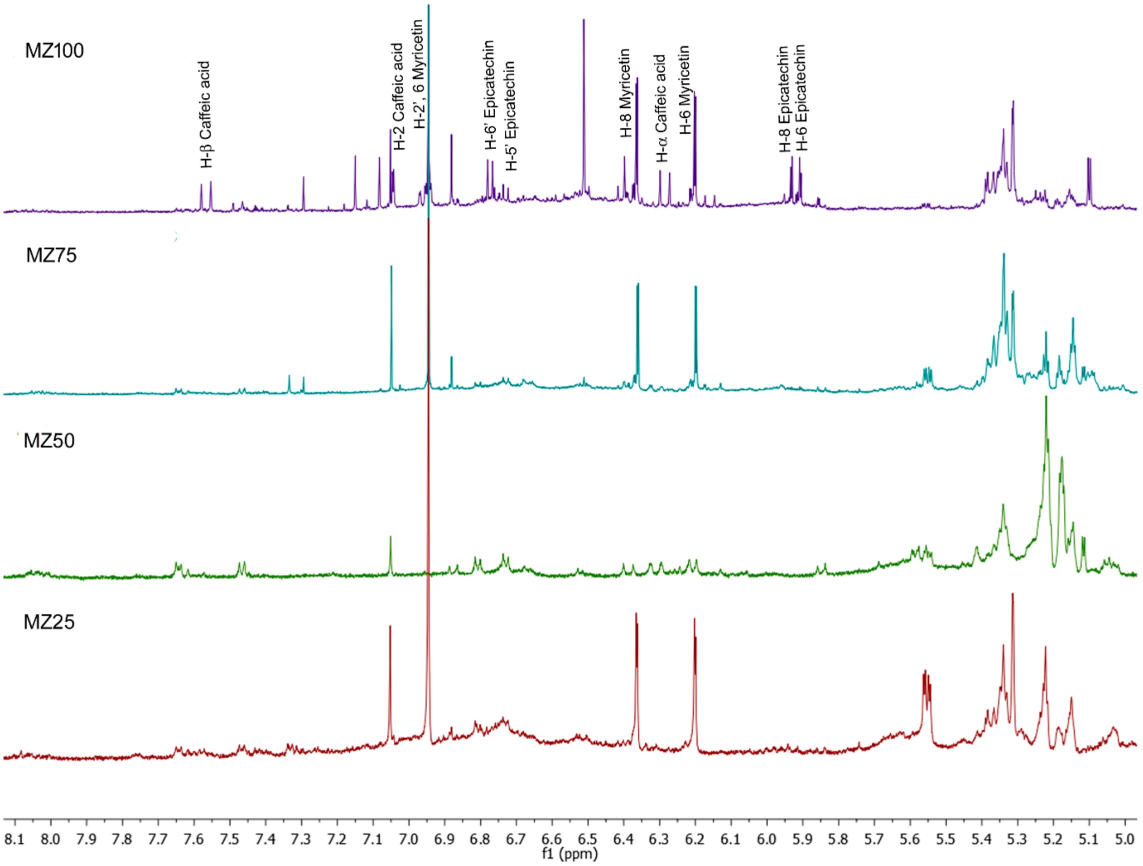

Figure 1. Representative ^{1}H-NMR profiles (Region δ 5.0 to 8.1) of the MeOH extracts from *M. zapota* leaves dried at the different temperatures (T = 25, 50, 75 and 100° C) showing the characteristic resonances from the detected metabolites.

References

1. Zhong, X.N.; Otsuka, H.; Ide, T.; Hirata, E.; Takushi, A.; Takeda, Y. Three flavonol glycosides from leaves of Myrsine seguinii. *Phytochemistry* **1997**, *46*, 943–946.
2. Usman, A.; Thoss, V.; Nur-e-Alam, M. Isolation of Taxifolin from Trichilia Emetica Whole Seeds. *Am. Sci. Res. J. Eng. Technol. Sci.* **2016**, *21*, 77–82.
3. Jeong, C.H.; Jeong, H.R.; Choi, G.N.; Kim, D.O.; Lee, U.; Heo, H.J. Neuroprotective and anti-oxidant effects of caffeic acid isolated from Erigeron annuus leaf. *Chin. Med.* **2011**, *6*, 25.

© 2019 by the authors. Licensee MDPI, Basel, Switzerland. This article is an open access article distributed under the terms and conditions of the Creative Commons Attribution (CC BY) license (http://creativecommons.org/licenses/by/4.0/).
